# Transcriptional cross-activation between toxin-antitoxin systems of *Escherichia coli*

**DOI:** 10.1186/1471-2180-13-45

**Published:** 2013-02-21

**Authors:** Villu Kasari, Toomas Mets, Tanel Tenson, Niilo Kaldalu

**Affiliations:** 1 Institute of Technology, University of Tartu, Nooruse 1, 50411, Tartu, Estonia

**Keywords:** Toxin-antitoxin systems, Transcriptional regulation, Regulatory network, mRNA stability, Persisters

## Abstract

**Background:**

Bacterial toxin-antitoxin (TA) systems are formed by potent regulatory or suicide factors (toxins) and their short-lived inhibitors (antitoxins). Antitoxins are DNA-binding proteins and auto-repress transcription of TA operons. Transcription of multiple TA operons is activated in temporarily non-growing persister cells that can resist killing by antibiotics. Consequently, the antitoxin levels of persisters must have been dropped and toxins are released of inhibition.

**Results:**

Here, we describe transcriptional cross-activation between different TA systems of *Escherichia coli*. We find that the chromosomal *relBEF* operon is activated in response to production of the toxins MazF, MqsR, HicA, and HipA. Expression of the RelE toxin in turn induces transcription of several TA operons. We show that induction of *mazEF* during amino acid starvation depends on *relBE* and does not occur in a *relBEF* deletion mutant. Induction of TA operons has been previously shown to depend on Lon protease which is activated by polyphospate accumulation. We show that transcriptional cross-activation occurs also in strains deficient for Lon, ClpP, and HslV proteases and polyphosphate kinase. Furthermore, we find that toxins cleave the TA mRNA *in vivo*, which is followed by degradation of the antitoxin-encoding fragments and selective accumulation of the toxin-encoding regions. We show that these accumulating fragments can be translated to produce more toxin.

**Conclusion:**

Transcriptional activation followed by cleavage of the mRNA and disproportionate production of the toxin constitutes a possible positive feedback loop, which can fire other TA systems and cause bistable growth heterogeneity. Cross-interacting TA systems have a potential to form a complex network of mutually activating regulators in bacteria.

## Background

Bacterial toxin-antitoxin (TA) systems are complexes of a stable toxic- or growth-arresting factor and its unstable inhibitor [1,2]. They are diverse, abundant in all bacteria, except a few intracellular parasites, and are found in many archaea [3-6]. On the basis of their ubiquity and diversity, we can assume that regulation by TA must be common and beneficial in a wide range of microorganisms. However, their role in bacterial physiology is unclear [7,8], in part due to redundancy [9]. They were first discovered in plasmids and characterized as addiction systems, which are responsible for post-segregational killing [10]. However, because of its high cost to the host, such a stability mechanism is used only in rare cases [11]. Chromosomal TA loci were found thanks to full genome sequencing [4] and were demonstrated to be functional, expressed at significant levels, and activated by various stressful conditions, particularly by amino acid starvation [12-15].

Our current study focuses on type II TA systems. In this group, both the toxin and the antitoxin are proteins, which are encoded by adjacent co-transcribed genes. In a growing cell, toxins are neutralized by tightly bound antitoxins. Antitoxins are degraded by proteases much more quickly than toxins, and if antitoxin production stops, toxins target vital functions of the producer through diverse mechanisms. Many toxins (e. g. RelE, MazF, YafQ, HigB, HicA, MqsR) are endoribonucleases and inhibit protein synthesis through cleavage of free or ribosome-bound mRNA [16-21]. MazF also cleaves 16S rRNA [22] and VapC endonucleases of enteric bacteria cleave initiator tRNA [23]. Another group of toxins (CcdB, ParE) interferes with DNA gyrase [24,25], whereas HipA is a protein kinase [26,27], and zeta toxins (PezT) inhibit cell wall synthesis [28]. Activation of toxins causes growth inhibition and dormancy that may be transient [29] but in some circumstances is irreversible and leads to cell death [28,30-32].

Besides direct protein-protein interaction, antitoxins regulate toxin activity at the level of transcription. Antitoxins are DNA-binding proteins and specifically repress transcription of their own TA operons both alone and, even more effectively, in complexes with their cognate toxins. Degradation of an antitoxin causes de-repression of the TA promoter [33] and allows the toxin activity to be detected indirectly by measurement of transcript levels. Gerdes and colleagues have demonstrated fine-tuning of transcription by the toxin:antitoxin ratio for the RelBE system [34,35]. The RelB antitoxin in excess of the RelE toxin promotes formation of the RelB:RelE (2:1) complexes that bind to the operator sites and repress transcription. RelE toxin in excess promotes formation of the ReB:RelE (2:2) complexes that are unable to bind DNA [36]. As a result, over-expression of RelE causes substantial increase in the *relBE* mRNA level. These authors suggested that such transcriptional regulation by the T:A ratio is commonplace for TA loci [35] and demonstrated it recently for VapBC [37]. Importantly, the levels of TA mRNAs were increased in cell populations enriched for persisters, thereby linking TA systems to antibiotic susceptibility [38,39]. Persisters are transiently dormant bacteria that remain non-dividing under growth-supporting conditions and are not killed by bactericidal antibiotics [40]. TA systems, by their very nature, may be primarily responsible for persister formation. Mutations that increase toxicity of the TA toxins were shown to increase the frequency of persisters and cause high persistence phenotypes [41,42]; and deletion of the *yafQ* toxin significantly decreased persister frequency in *E. coli* biofilms [43]. A recent study reports that successive deletion of 10 endoribonuclease-encoding TA loci progressively reduced the level of persisters while single deletions of TA systems had no effect on persister frequency in planktonic *E. coli*[44]. Hence, it is extremely important to consider redundancy and possible cross-talk when we study TA-related phenotypes, because most bacterial genomes contain multiple TA loci.

In the current study we found that uninhibited toxins can activate transcription of the other TA operons. Cleavage of these transcripts by endoribonuclease toxins adds another layer of complexity. Reciprocal transcriptional de-repression and transcript cleavage predict that toxin-antitoxin systems have a potential to form a complex network of regulators that controls growth and dormancy of bacteria.

## Results

### Uninhibited toxins can activate other toxin-antitoxin systems

**Figure 1:**
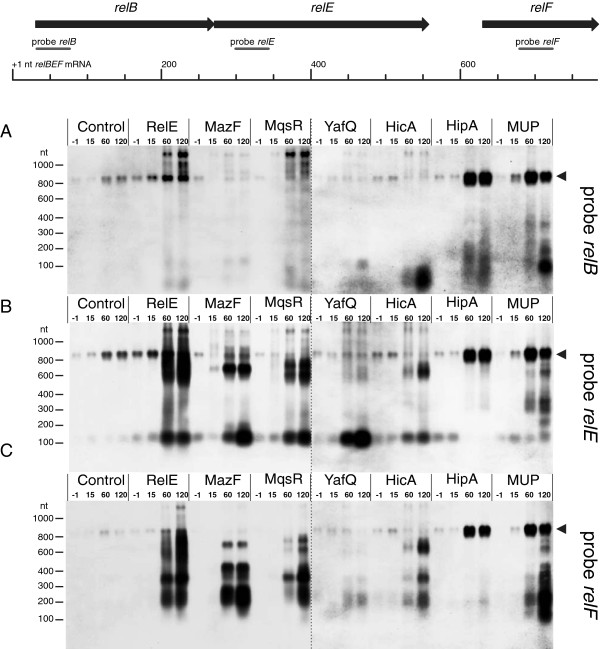
**Northern analysis of *relBEF *transcription in response to expression of different toxins.** Cultures of BW25113 contained plasmids for toxin and antitoxin expression. Toxins were induced and RNA was extracted at timepoints −1(before induction), 15, 60, and 120 min; 10-μg aliquots were subjected to electrophoresis, transferred to a membrane, and hybridized with oligoprobes relB (**A**), relE (**B**), and relF (**C**). Localization of the hybridization probes is shown on the map of the *relBEF* operon and the full-length *relBEF* transcript is marked by arrowhead (◄). Cultures of toxin over-expression contained the following plasmids: RelE - pVK11; MazF - pSC3326 and pSC228; MqsR - pTX3 and pAT3; YafQ - pBAD-*yafQ* and pUHE-*dinJ*; HicA - pMJ221 and pMJ331; HipA - pNK11 and pNK12. Control cultures contained the empty vectors pBAD33 and pOU82. Mupirocin (MUP) was added as a positive control for transcriptional activation of *relBEF*.

**Figure 2:**
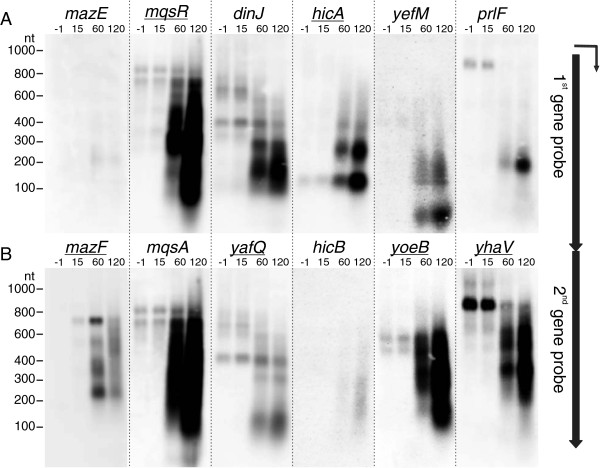
**Transcription of TA operons in response to expression of RelE.** Production of RelE was induced in cultures of BW25113 bearing plasmids pKP3035 and pKP3033. RNA extracted at timepoints −1 (before induction), 15, 60, and 120 min was subjected to northern analysis using oligoprobes complementary to the mRNAs of different toxins (underlined) and antitoxins. Panel **A** refers to the first and panel **B** to the second gene of the TA operon.

Excess of a toxin has been shown to destabilize binding of the toxin-antitoxin complex to operator DNA and to activate transcription of its own operon [35]. To test whether toxins can activate transcription of other TA operons, we measured the transcription of *relBE* in response to ectopic expression of toxins MazF, MqsR, YafQ, HicA, and HipA by northern hybridization (Figure 1). Since the *relBE* genes are co-transcribed with the downstream *relF*[45], which encodes a *hok*-like toxin targeted against the inner membrane [46], we analyzed the transcription of the full *relBEF* operon. In a reverse experiment, we over-expressed RelE and monitored the transcription of several chromosomal TA operons (Figure 2). Amino acid starvation is known to upregulate *relBEF* transcription [14] and was induced by addition of mupirocin (MUP) [47] as a positive control. Ectopic expression of RelE served as an additional positive control for activation of *relBEF* transcription whereas synonymous substitutions were introduced into the plasmidal *relE* sequence (Additional file 1: Table S2; primer relE-XbaUP) to enable unambiguous detection of the chromosomal *relE* transcript. Active RelE toxin could be expressed from the altered gene (Additional file 1: Figure S1) and the plasmidal transcript was not detectable in the Δ*relBEF* strain, showing that our hybridization probes are specific and do not cross-hybridize (Additional file 1: Figure S3A,B,C lanes 1,2). Toxins were induced in log phase cultures and concomitant measurements of optical density confirmed growth inhibition in all cultures tested (Additional file 1: Figure S1). Samples for RNA isolation were collected before induction (−1 min) and during a two hour time-course post-induction (15, 60 and 120 min); mRNA of the chromosomal TA operon was analyzed by northern hybridization using DNA oligoprobes complementary to *relB*, *relE*, and *relF* (Figure 1; Additional file 1: Table S2).

As shown in Figure 1, we indeed saw a clear cross-activation of *relBEF* in response to all toxins tested except YafQ. Induction of RelE, MazF, MqsR, HicA and HipA conferred a clear increase in the *relBEF* mRNA level in an hour. Use of three separate probes revealed, however, that different mRNA species pile up in response to different toxins. Before induction and 15 min after, all three probes – relB, relE and relF – detected a transcript of the same size corresponding to the full-length mRNA of the operon [45], as confirmed later by primer extension mapping of the 5^′^ end (Additional file 1: Figure S4). Only after MazF expression a shorter transcript, a putative cleavage product, could be detected at the 15 min time point using relE probe (Figure 1B). At later time points, hybridization with relB (Figure 1A) and relE (Figure 1B) probes gave different signals: in response to induction of MazF, MqsR, and HicA we saw cleavage of the full-length mRNA and massive accumulation of the toxin-encoding part, while the antitoxin-coding portion could not be detected and was apparently degraded (Figure 1A,B). Such cleavage and accumulation of the toxin portion also occurred in response to RelE. Hybridization with relF probe revealed additional cleavage, both within *relE* and downstream, in response to expression of all these toxins, and the *relF* part accumulated as the most abundant portion of the *relBEF* transcript (Figure 1C). Also, some transcripts larger than the full *relBEF* mRNA appeared, particularly after induction of RelE and MqsR. Production of HipA, which is not a ribonuclease, conferred strong induction of full-length *relBEF* mRNA but cleavage and uneven accumulation of different mRNA fragments could not be seen. MUP treatment produced overproduction of the full *relBEF* mRNA as well as accumulation of some cleavage products. Production of YafQ did not lead to a clear cross-activation of *relBEF* transcription. However, relE probe showed accumulation of a short RNA fragment in response to this toxin. It is possible, that transcription of the operon is activated by YafQ but the transcript is degraded to small fragments. Clearly, these fragments cannot serve as templates for synthesis of RelE and, therefore, functional cross-activation does not occur. Modest induction of *relBEF* with no cleavage was evident in the 1h and 2h samples of control cultures, lacking artificial production of any free toxin. We have to consider that, at this stage, the control cultures were approaching stationary phase, and induction of toxin-antitoxin modules has been described in similar conditions [48].

Probes complementary to *yiaF* and *rpsS* were used for control because the levels of transcription of these genes did not differ between log phase cells and the ampicillin-refractory non-growing subpopulation, where TA operons were highly expressed [38]. *rpsS* is a part of the large S10 ribosomal protein operon with an estimated transcribed length of 5181 bp [49]; *yiaF* (711 bp ORF) encodes for a putative membrane protein of unknown function; it is located between genes pointing in the opposite direction and must form a single-gene operon. The control mRNAs were not induced by toxins (Additional file 1: Figure S2B,C). After induction of toxins, the *yiaF* transcript was degraded without accumulation of any stable fragments. (Additional file 1: Figure S2B). Surprisingly, mupirocin initially induced transcription of *yiaF* whereas the level of the transcript dropped after longer incubation (Additional file 1: Figure S2B). The S10 transcript was degraded as well. Some accumulating stable fragments of the S10 transcript were detectable after MazF, RelE and MqsR production (Additional file 1: Figure S2C).

To be sure that the accumulating RNA fragments, which correspond to the 3^′^ portion of the *relBEF* mRNA, are not initiated from toxin-inducible cryptic promoters within the operon, we deleted the promoter of the *relBEF* operon. In the promoterless BW25113 ΔP_*relBEF*_ strain, we did not see induction of the *relBEF* mRNA nor the characteristic accumulation of its 3^′^ portion (Additional file 1: Figure S3). We still saw a transcript that could be detected by the relE and relF probes (Additional file 1: Figure S3B,C) but the level of this transcript did not depend on the RelE production. It might be initiated from a constitutive promoter that was newly created by deletion of P_*relBEF*_. Transiently induced smear of RNA that was detected in BW25113 ΔP_*relBEF*_ with the relB probe (Additional file 1: Figure S3A, lanes 6 and 7) is transcribed from the RelB-expression plasmid pKP3033. That is the reason why we omitted this plasmid when we studied induction of *relBEF* in response to RelE (Figure 1, Additional file 1: Figure S3, lanes 8–11). Thus, we can be sure that the shorter transcripts that massively pile up in response to toxins are indeed cleavage products and are initiated at the genuine *P*_*relBEF*_ promoter.

Next, we tested whether over-production of the toxin RelE activates other toxin-antitoxin genes in the chromosome. The northern hybridization results show strong induction of the *mqsRA*, *mazEF*, *dinJ-yafQ*, *hicAB, yefM-yoeB*, and *prlF-yhaV* TA systems (Figure 2). Similarly to *relBEF*, the induced transcripts were cleaved and the toxin-encoding parts seem to accumulate preferentially while the antitoxin-coding parts are more effectively degraded. That appears to be true irrespective of whether the toxin is encoded by the first (*mqsRA*, *hicAB*) or the second (*mazEF*, *yefM-yoeB*, *prlF-yhaV*) gene of the operon (Figure 2). Reliable testing of this phenomenon requires characterization of the cleavage products and additional experiments in the future.

Additional experiments indicated that transcriptional cross-activation of TA operons does not occur between all possible TA combinations. Northern hybridization using mqsR probe showed that overproduction of MazF and HicA does not induce the *mqsRA* promoter while YafQ and HipA induce it (data not shown), as well as RelE (Figure 2).

### Activation of *mazEF* by amino acid starvation is dependent on *relBE*

**Figure 3:**
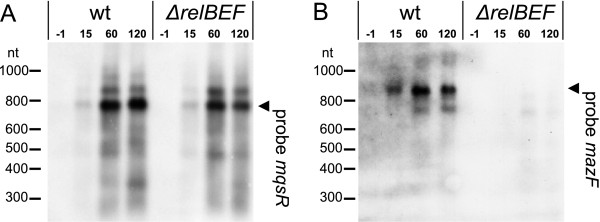
**Transcription of *mqsRA *and *mazEF *operons in response to amino acid starvation.** Mupirocin (MUP) was added to cultures of BW25113 (wt) and BW25113 ∆*relBEF* to inhibit isoleucine tRNA synthetase and induce stringent response. RNA was extracted at timepoints −1 (before addition of MUP), 15, 60, and 120 min; 10-μg aliquots were subjected to northern blotting and hybridized with probes mqsR (**A**) and mazF (**B**). The full-length *mqsRA* and *mazEF* transcripts are marked by arrowheads (◄). A longer *mqsRA* transcript can be seen above the marked band and has been described previously [59].

We wanted to test whether TA cross-activation happens also during natural physiological stresses. Amino acid starvation has been shown to induce transcription of the *relBE*[14] and *mazEF*[17] genes. We induced amino-acid starvation by addition of mupirocin to the cultures of BW25113 (wild type) and BW25113Δ*relBEF*. Northern analysis indicated that transcription of *mazEF* is upregulated only in wild type bacteria and not in the *relBE* deficient strain (Figure 3B). Transcription of *mqsRA*, the other TA operon that we tested, was induced in both strains, independently of the RelBE system (Figure 3A). Thus, RelBE system activates another TA system, MazEF, in response to amino acid shortage. This evidences that TA cross-activation is not a mere artifact of toxin overexpression but occurs as a part of a real physiological response.

### Cross-activation occurs in *lon*, *ppk*, *clpP*, and *hslV* deficient strains

**Figure 4:**
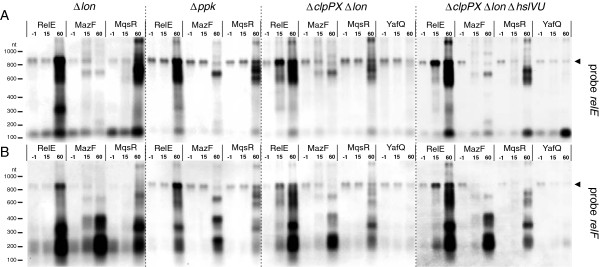
**Transcriptional activation of *relBEF *in protease- and polyphosphate kinase deficient strains.** Cultures of BW25113 ∆*lon*, BW25113 ∆*ppk*, BW25113 ∆*clpPX*∆*lon*, and BW25113 ∆*clpPX*∆*lon*∆*hslVU* contained pVK11 (RelE), pSC3326 (MazF), pTX3 (MqsR), or pBAD-*yafQ* plasmid for toxin expression. Toxins were induced and RNA was extracted at timepoints −1 (before induction), 15 and 60 min; 10-μg aliquots were subjected to northern blotting and hybridized with probes relE (**A**) and relF (**B**). The full-length *relBEF* transcript is marked by arrowhead (◄).

Since it is widely accepted that TA loci are activated by proteolytic degradation of antitoxins, we tested whether transcriptional cross-activation is affected by Lon, ClpP or HslV proteases. Besides, we tested the requirement of polyphospate, which has been shown to activate Lon [50]. We expressed RelE, MazF, and MqsR toxins in BW25113 strain lacking *lon* or *ppk*, which encode for Lon and polyphosphate kinase, respectively, and observed chromosomal *relBEF* transcript by northern hybridization using probes relE and relF (Figure 4). Deletion of *lon* or *ppk* did not abolish cross-induction of *relBEF* by MqsR, and as seen on relF probed blot (Figure 4B), by MazF. We further tested *relBEF* activation in a double-knockout strain lacking Lon and ClpP, and a triple-knockout lacking Lon, ClpP and HslV proteases. Again, expression of MazF and MqsR obviously induced *relBEF* in the strains deficient for multiple proteases (Figure 4). Accumulating RelE-, MazF- and MqsR- specific cleavage intermediates produced similar patterns in all tested strains (Figure 1B,C, Figure 4). Production of YafQ did not cause a clear activation of *relBEF* transcription in the protease-deficient strains, similarly to the wt strain. Accumulation of a small fragment hybridizing to the relE probe can be detected in the Δ*clpPX*Δ*lon*Δ*hslVU* strain (Figure 1B, Figure 4A). Ectopic production of RelE induced transcription of chromosomal *relBEF* in all strain backgrounds, as expected. Essentially, we can conclude that cross-activation of TA transcription occurs also in *lon*^*-*^, *ppk*^*-*^, *clpPX*^*-*^*lon*^*-*^, and *clpPX*^*-*^*lon*^*-*^*hslVU*^*-*^ backgrounds.

### Cleavage of the relBEF mRNA *in vivo*

**Figure 5:**
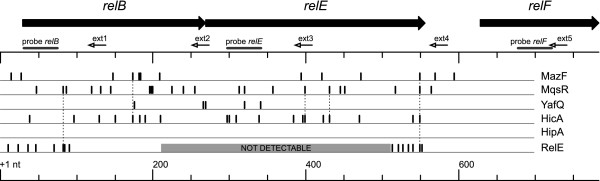
**Cleavage of the *relBEF *mRNA *in vivo*.** The same RNA samples that were analyzed by northern blotting (Figure 1) were subjected to primer extension analysis shown in (Additional file 1: Figure S4). Detected 5′ ends, localization of the extension primers and hybridization probes are mapped on to the *relBEF* operon. Dotted lines mark cleavage sites that occur in response to several over-produced toxins. The gray bar indicates the region where detection of the cleavage sites in the *relBEF* mRNA was impossible owing to the plasmidal *relE* mRNA transcribed from pVK11.

To characterize the *in vivo* cleavage of *relBEF* mRNA in more detail, we mapped the 5^′^ ends of the cleavage products using primer extension analysis (Figure 5, Additional file 1: Figure S4, Table S3). As seen in Figure 5, the cleavage sites in the mRNA, which was purified from the cells with over-expression of the nucleases MqsR and HicA, are distributed all over the operon. Several specific cutting sites of the MazF nuclease are found in the RelB-encoding part. No cleavage is detected in response to production of the protein kinase HipA, as expected. Most of the cutting sites were unique for each toxin indicating that the cleavage *in vivo* was a result of primary activity of the over-produced toxin. RNA from MazF and MqsR over-expression samples was mostly cleaved at the specific cutting sites of these toxins, i.e. ACA [51] and GCU [16]. However, several unique cleavage sites in the MazF and MqsR over-expression samples do not contain these sequences and might be generated by unidentified ribonuclease(s), possibly cross-activated toxins (Additional file 1: Table S3). We also observed that not all ACA and GCU sequences were cleaved in the *relBEF* mRNA by MazF and MqsR, respectively. As before [19], the cleavage preferences of HicA could not be identified.

To confirm our notion of TA cross-activation, we hoped to see some cleavage hotspots. At those sites, strong cleavage by an overproduced toxin occurs at its specific cutting sequence (e.g. ACA in the case of MazF). Cleavage at the same site in response to expression of another toxin would indicate activation of the primary cutter by the over-produced toxin. We tested possible cross-activation at three of these sites. At position 174 (ˇACA), the *relBEF* transcript is cut by MazF and in response to the over-produced HicA. The MqsR-specific cleavage sites at positions 399 (GCˇU) and 431 (GˇCU) are also cleaved in the samples from HicA over-production (Additional file 1: Figure S4). We found that these cuts were not due to the activation of MazF and MqsR, since they occurred in RNA extracted from the BW25113Δ*mazEF* and BW25113Δ*mqsRA* cells (data not shown). ChpBK, a homolog of MazF with similar but relaxed sequence specificity [52] may be accountable for the cleavage at 174 (ˇACA).

### The cleavage products of relBEF mRNA can be translated into proteins

The toxin-encoding parts of the TA transcripts seem to be generally more stable than the antitoxin-encoding parts and accumulate after cleavage (Figures 1, 2). If the toxin open reading frame (ORF) on these cleavage products is intact and translated into a functional protein, the T:A balance must be shifted towards toxin followed by more cleavage, cross-activation of other TA systems, and inhibition of protein synthesis. That creates the possibility of a positive feedback circuit and even a network of them. A positive autoregulatory loop, in turn, could explain the bistability of bacterial growth observed in response to toxin expression [53,54].

**Figure 6:**
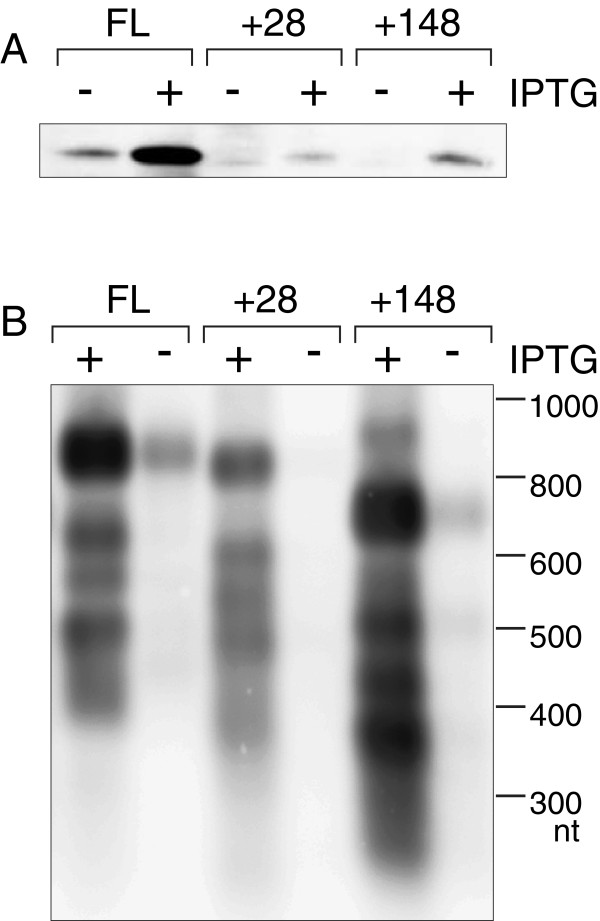
**RelE toxin can be translated from mRNAs resembling the accumulating cleavage fragments of the *relBEF *transcript.** Cultures of BL21(DE3) contained plasmid pNK31 for T7 expression of an mRNA starting at the 5′end of the full-length (FL) *relBEF* transcript; pNK32 for expression of an mRNA starting at the position + 28; and pNK33 for expression of an mRNA with disrupted *relB* open reading frame starting at position +148. Expression of T7 RNA polymerase was induced for 1 h by adding 1mM IPTG. Control cultures were grown without IPTG. Total protein lysates were analyzed for expression of RelE(R81A/R83A)-C-His using western blotting (**A**), and RNA expression was analyzed by northern hybridization using oligoprobe relE (**B**).

To test whether proteins are translated from the cleaved *relBEF* mRNA, we used the T7 promoter for expression of two transcripts, which begin at the sites of MazF-inflicted cleavage, at positions +28 and +148 from the 5^′^ end of the full-length transcript, and extend downstream of the *relE* ORF. The +28 RNA starts immediately upstream of the *relB* ORF (Additional file 1: Figure S4). Thus, the *relB* ORF is leaderless and lacks the upstream untranslated region with the ribosome binding site (RBS). The +148 RNA starts in the middle of the *relB* ORF. To allow RelE to be detected, we added the His6 tag to the C-terminus of the toxin and introduced substitutions R81A and R83A, which reduce its toxicity [55]. Expression of these RNAs in BL21(DE3) resulted in production of the toxin RelE(R81A/R83A)-C-His, although in smaller quantities than from the control transcript with the intact 5^′^ end (Figure 6). Thus, the accumulating cleavage products of TA mRNA can be translated into proteins, although less effectively than full transcripts with intact RBS in front of *relB*. Reduced translation of the downstream *relE*(R81A/R83A)-C-His open reading frame in shorter transcripts suggests that *relE* lacks its own RBS and it is produced due to translational coupling of *relBE* genes. Translational coupling of polycistronic TA mRNA has been demonstrated previously for *parD* (*kis-kid*) of plasmid R1 [56].

### Transient expression of toxins can induce bistability of growth

Production of toxins causes an extensive rearrangement of bacterial physiology. It can inflict dormancy and antibiotic tolerance [57] if the toxin level exceeds a threshold [54]. Fluctuations in toxin levels above and below the threshold have been used to explain the coexistence of dormant and growing cells in a population [54]. The possibility of positive feedback by the generation and selective buildup of the toxin-encoding mRNA fragments may explain this heterogeneity in growth. Therefore, we wanted to evaluate the recovery of single bacteria and test possible growth heterogeneity after over-production of a toxin and the resulting activation of the chromosomal TA loci. We monitored growth resumption by individual cells using dilution of previously synthesized green fluorescent protein (GFP) [58]. The plasmid pTM11 was inserted into the chromosome of BW25311 to allow IPTG-inducible GFP to be expressed, and this strain was transformed with plasmids for L-arabinose-inducible production of toxins RelE, MazF, MqsR and HipA. Expression of GFP was induced for 2.5 h; thereafter, the cells were transferred into medium containing L-arabinose to induce the toxins. After 90 min, the growth medium was changed again to shut down toxin synthesis and allow recovery (Additional file 1: Figure S5). Analysis of the bacterial GFP content by flow cytometry (Additional file 1: Figure S6) showed that after temporary expression of RelE and HipA the bacteria resumed growth rather uniformly, while after expression of MazF and MqsR a subpopulation started to grow with a delay. Thus, expression of these toxins created bistability in a population. Most importantly, all bacteria resumed growth after the transient expression of toxins. Although inhibition by MazF and MqsR was apparently stronger and induced growth heterogeneity, it did not generate a subpopulation of persistently non-dividing bacteria (Additional file 1: Figure S6).

## Discussion

### Mutual cross-activation of TA systems

**Figure 7:**
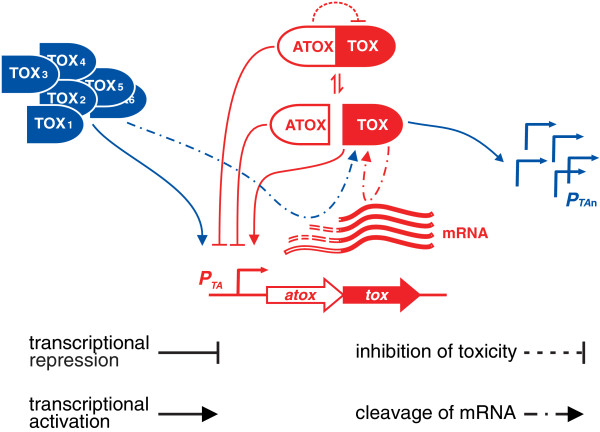
**Toxin-antitoxin systems are subject to both auto- and cross-regulation.** Cognate regulatory interactions are in red and non-cognate interactions are in blue. According to the established model, cognate antitoxin and toxin, which are encoded by co-transcribed genes, form a tight complex and antitoxin inhibits the toxin through direct protein-protein interaction. Antitoxin, both alone and in complex with the toxin, binds to the operator DNA and auto-represses transcription of the TA operon. Free toxin in excess disrupts this DNA-protein interaction and induces transcriptional de-repression. We show that transcription of TA genes can be induced also by non-cognate toxins. Moreover, cleavage of the TA mRNA by both cognate and non-cognate toxins results in accumulation of the toxin-encoding mRNA fragments. Translation of these fragments can lead to accumulation of free toxin.

Sequential or simultaneous activation of different TA systems has been reported elsewhere. Transcription of several TA operons was induced in the persister-enriched subpopulation [38,39]. Amino acid starvation in *E. coli* activated both RelE and MazF (ChpAK) [14,17]. We observed induction of the *mqsRA* system in response to HipA activation [59], whereas overproduction of MqsR induced transcription of *relBE* and *relF*(*hokD*) [60]. Also, ectopic expression of VapC toxins originating from *Salmonella* and *Shigella* activated YoeB [61] and production of the Doc toxin activated RelE in *E. coli*[62]. Here, we show that overexpression of several toxins can activate transcription of the other TA operons. Since toxins and TA operons in this study present a random sample, such cross-interactions might be common and be the rule rather than the exception. Consequently, TA systems have a potential to form a cross-activation network, which operates at the transcriptional level (Figure 7). The presence of such network versus lone and uncoordinated TA systems must have an impact on TA activity during the stress response and setup of dormancy.

Induction of the chromosomal *relBEF* in response to the ectopically produced RelE can be explained by conditional cooperativity (dependence of transcriptional regulation on the T:A ratio) [35]. However, according to our current knowledge, such mechanism is not applicable to cross-induction. Activation of YoeB by VapC depended on Lon protease [61]. Also, Lon was required for induction of TA operons in response to amino acid starvation and chloramphenicol [14,17,18,61]. Our experiments do not provide a solid support for the role of Lon and ClpP in cross-regulation between TA systems of *E. coli* (Figure 4). Since the cross-induction was present in the knock-out strains, an additional, Lon-, ClpP-, HslV-, and polyphosphate-independent mechanism of regulation must be involved. Unlocking this mechanism remains a task for future studies. The simplest explanation to activation of TA systems would be depletion of antitoxins. It must inevitably happen when protein synthesis decreases. That predicts nonselective induction of all TA operons in response to inhibition of translation, no matter if it is caused by starvation or artificial production of a toxin. Requirement of *relBE* for transcriptional activation of *mazEF* during amino acid starvation (Figure 3) contradicts this prediction as well as the lack of *mqsRA* induction in response to overproduction of MazF and HicA (data not shown). An option for a mechanism of cross-activation is positive feedback regulation due to selective accumulation of toxin-encoding fragments upon mRNA cleavage. As we saw, after cleavage by overproduced toxin, the antitoxin-encoding RNA fragments are rapidly degraded while the toxin-encoding fragments may serve as templates for translation of toxin. Different toxins produce different cleavage products. That can potentially explain why they cause unequal level of trans-activation when overproduced.

Another intriguing issue of TA cross-reaction is the possible cross-inhibition due to non-cognate interactions. Some authors report such cross-reactions [63-68] while others have tested but not found them [69,70]. As a part of this study, we examined non-cognate inhibition between *E. coli* toxins and antitoxins of the RelBE, MazEF, MqsRA, and HipBA systems *in vivo*. In this attempt, we run into a previously described phenomenon that may become a source of erroneous results. If toxins are expressed from the arabinose-inducible *P*_BAD_ promoter and antitoxins from an IPTG-inducible promoter, it is important to consider that IPTG inhibits *P*_BAD_ directly [71]. When we used an expression vector that encoded for the IPTG-insensitive C280* version of AraC transcriptional activator, we could not see any cross-inhibition. Based on that, a recent report on functional non-cognate TA interactions in *Mycobacterium tuberculosis*[67] may require retesting.

### Selective targeting of mRNA by toxins as a mechanism of gene regulation

In the current study, we found that the cleavage products produced by TA toxins differ in stability. Selective targeting of mRNAs by endoribonucleolytic toxins and different stabilities of the resulting cleavage products may constitute another layer of gene regulation in the bacterial stress response. Differences in half-life and translational efficiency of mRNA cleavage products, along with generation of a pool of ribosomes lacking the anti-Shine-Dalgarno sequence (as shown for MazF [22]), could profoundly affect the proteome composition. An example of such an effect is the occurrence of a MazF-resistant protein pool in *E. coli*[72]. The accumulation of toxin-encoding mRNA fragments may have potential use as a marker of toxin activation in studies of stressed and non-growing bacteria. Increase of the T/A ratio may possibly trigger a positive feedback loop consisting of transcriptional activation of the TA operon, successive cleavage of the TA transcript, buildup of the toxin-encoding mRNA fragments, and translation of them, shifting the T/A balance (Figure 7). Thus, it can be related to TA-linked growth heterogeneity in bacterial populations (Additional file 1: Figure S6) [38,39,54].

## Conclusions

The main finding of this study is that bacterial toxin-antitoxin systems affect mutually each others’ expression and activity (Figure 7). We show that overexpression of one toxin can activate transcription of the other TA operons. Toxins with endoribonuclease activity add another layer of complexity to these interactions. They cleave TA mRNA, which is followed by degradation of the antitoxin-encoding RNA fragments and accumulation of the toxin-encoding fragments. We show that these accumulating mRNA fragments can be translated to produce more toxin.

Most of bacteria have many different TA systems. Although their function is debatable, many TA toxins have similar activity and the inhibitory effect on bacterial cells is common to all of them. Therefore, an important question is whether TA systems are redundant or not. Another intriguing issue is whether different TA systems are functionally connected and do cross-talk [44,70].

Here we over-expressed toxins to show that TA systems have a potential to form a network of cross-reacting regulators in *E. coli*. We found an example of such cross-reaction, which occurs without artificial overexpression: the *relBE*-dependent transcriptional activation of *mazEF* during amino acid starvation. It remains a rather difficult task to identify the mechanism(s) of TA cross-activation. Currently we know that cross-activation is not dependent on major proteases Lon, ClpP, and HslV. Also, it cannot be a self-evident outcome of antitoxin shortage since we know examples where shutdown of protein synthesis does not activate a TA promoter.

## Methods

### Bacterial strains, plasmids and growth conditions

All strains and plasmids are listed in Additional file 1: Table S1. Conditions of bacterial cultivation and construction of strains and plasmids are described in Additional file 1: Supporting information.

### Northern hybridization

Procedures for blotting and hybridization are described in [59]. *E. coli* BW25113 was transformed with two plasmids, one bearing an antitoxin gene and the other bearing a toxin gene. Cultures containing the empty vector plasmids pBAD33 and pOU82 were used for negative controls. When bacteria contained plasmids for toxin expression, the LB medium for overnight cultures was supplemented with 0.2% glucose and 50 μM IPTG (for HicA with 1mM L-arabinose). Overnight cultures were diluted 1000-fold into 200 ml of LB and grown to OD_600_ ≈ 0.2 (for ~ 2.5 h). To induce toxins, 1 mM L-arabinose, 1 mM IPTG (for HicA) or 30 μg ml^−1^ mupirocin was added. Overnight cultures of BW25113 Δ*relBEF* and BW25113 Δ*P*_*relBEF*_ containing plasmids were diluted into LB supplemented with 0.2% glucose and 50 μM IPTG; at OD_600_ ≈ 0.2, bacteria were collected by centrifugation (5 min, 5000g, at 20°C) and resuspended in prewarmed LB supplemented with 1 mM L-arabinose. Total RNA was extracted using two different protocols: in Figures 2, 6 and S3 we used Trizol reagent [59] and in all other experiments we used hot phenol (for details see Additional file 1: Supporting information). Samples of total RNA (10 μg) were subjected to electrophoresis on denaturing gels. The DNA oligoprobes used for hybridization are listed in Table S2 (Additional file 1). For re-hybridization, the membranes were stripped by boiling for 2×10 min in 0.1% SDS, 5mM EDTA. Chemiluminescent signals were captured using ImageQuant RT ECL imager (GE Healthcare) and X-ray film (Agfa).

### Primer extension

RNA samples were collected as for northern blotting. Extension primers (Additional file 1: Table S2) were labeled with [γ^32^P]ATP by T4 polynucleotide kinase (Thermo Scientific) and purified with a Nucleotide Removal Kit (Qiagen). Total RNA (15 μg) was mixed with labeled primer and incubated at 75°C for 2 min followed by slow cooling for 25 min. Extension reactions were carried out at 44°C for 30 min using 200U of RevertAid^TM^ H minus reverse transcriptase (Thermo Scientific) and stopped with 10 μl of formamide loading buffer [73]. Reaction products were concentrated by ethanol precipitation before gel electrophoresis. DNA was sequenced using a Sequenase Version 2.0 Kit (USB Products, Affymetrics). A PCR product amplified using primers relBEFup and relFdwn, and treated with Exonuclease I and shrimp alkaline phosphatase (ThermoScientific), was used as the template for the sequencing reactions. Samples were analyzed by 7M urea-6% polyacrylamide gel electrophoresis.

### Protein electrophoresis and western blots

To prepare lysates, bacteria were grown to an OD_600_ of ~0.7 and expression of T7 RNA polymerase was induced for 1 h by adding 1mM IPTG. Control cultures were grown without IPTG. Bacteria were spinned down and lysed in Laemmli sample buffer. Proteins were separated by tricin–SDS–13% polyacrylamide gel electrophoresis [74]. For detection of the His6-tagged toxins, the proteins were electroblotted onto Hybond-ECL nitrocellulose membrane filters (GE Healthcare) and probed with nickel-activated horseradish peroxidase (HisProbe^TM^-HRP; Thermo Scientific).

### Growth resumption experiments

Overnight cultures were grown from fresh single colonies for 17–18 h in LB supplemented with 0.2% glucose and diluted 500-fold, into 3 ml of broth. After 2 h of incubation, 1 mM IPTG was added to initiate synthesis of green fluorescent protein (GFP). Expression of GFP was induced for 2.5 h. Then, cells were collected by centrifugation and transferred into LB supplemented with 0.2% L-arabinose to induce toxin synthesis. During the change of the medium, the culture was diluted 10-fold. After 90 min, the growth medium was changed to LB containing 0.2% glucose to stop the production of toxins, this time with 2-fold dilution. Starting from the induction of toxin synthesis, samples were taken for flow cytometry analysis and OD_600_ measurement. For flow cytometry analysis, the samples were mixed with an equal volume of 30% glycerol in PBS and stored at −80°C pending analysis. After dilution with sterile PBS, the samples were analyzed using an LSRII and a high-throughput sampler (BD) with a laser beam maximum wavelength of 488 nm. The results were analyzed by using FlowJo 7.2.1software.

### Reproducibility of experiments

All growth inhibition (Additional file 1: Figure S1) and growth resumption experiments (Additional file 1: Figure S5, S6) were repeated at least three times. All northern blot (Figures 1, 2, 3, 4, 6 Additional file 1: Figures S2, S3), primer extension mapping (Additional file 1: Figure S4) and *in vivo* translation experiments (Figure 6) were repeated at least twice with similar results. Typical results are presented for these experiments and for the FACS analysis of growth resumption (Additional file 1: Figure S6).

## Competing interests

The authors declare that they have no competing interests.

## Authors’ contributions

VK and NK designed the study, analyzed results and drafted the manuscript. VK performed the RNA analysis. TM performed flow cytometry, helped with the other experiments and provided suggestions about the manuscript. NK helped with the experiments. TT contributed to the study design, analysis and drafting of the manuscript. All authors have read and approved the manuscript.

## Supplementary Material

Additional file 1 **Supplemental experimental procedures. Figure S1. **Growth of the cultures used for extraction of RNA. **Figure S2. **Northern analysis of *yiaF* and *rpsS* transcription in response to expression of different toxins.**Figure S3. **Northern analysis of transcription of the *relBEF* operon lacking its native promoter in response to ectopic expression of RelE.**Figure S4. **Primer extension mapping of cleavage of the *relBEF* mRNA.**Figure S5. **Growth of bacteria for monitoring recovery from transient expression of toxins.**Figure S6. **Growth resumption after transient production of toxins.**Table S1. **Strains and plasmids used in this study.**Table S2. **Oligonucleotides used in this study.**Table S3. **Cleavage sites of *relBEF* mRNA in vivo. Click here for file
