# Supplementary material for: Transcriptional cross-activation between toxin-antitoxin systems of Escherichia coli
Source: BMC Microbiol. 2013 Feb 21;13:45. doi: 10.1186/1471-2180-13-45 (PMC3598666; doi:10.1186/1471-2180-13-45)
Supplement: Additional file 1 — Supplemental experimental procedures. Figure S1. Growth of the cultures used for extraction of RNA. Figure S2. Northern analysis of yiaF and rpsS transcription in response to expression of different toxins.Figure S3. Northern analysis of transcription of the relBEF operon lacking its native promoter in response to ectopic expression of RelE.Figure S4. Primer extension mapping of cleavage of the relBEF mRNA.Figure S5. Growth of bacteria for monitoring recovery from transient expression of toxins.Figure S6. Growth resumption after transient production of toxins.Table S1. Strains and plasmids used in this study.Table S2. Oligonucleotides used in this study.Table S3. Cleavage sites of relBEF mRNA in vivo. [file 1471-2180-13-45-S1.pdf]

Supporting Information

Transcriptional cross-activation between toxin-antitoxin systems of *Escherichia coli*

Villu Kasari, Toomas Mets, Tanel Tenson, and Niilo Kaldalu*
Institute of Technology, University of Tartu, Nooruse 1, 50411 Tartu, Estonia

Supplemental Experimental Procedures

Bacterial strains, plasmids, and growth conditions

The *E. coli* strains and plasmids used are listed in Table S1. Deletions Δ*relBEF*, ΔP_{relBEF} and Δ*clpPX*Δ*lon* were made using the method of Datsenko and Wanner [1]. The PCR products for Red-mediated recombination were generated using knockout primers listed in Table S2. Deletions were verified with knockout control primers (Table S2) in combination with primers k1, k2, and kt [1]. BW25113 Δ*hslVU*Δ*clpPX*Δ*lon* was constructed by P1 transduction of the Δ*clpPX*Δ*lon*::*kan* into BW25113 ΔhslVU::*FRT*. Single-copy integration of CRIM plasmid pTM11 into the *att*φ80 site of BW25113 chromosome was carried out, and integrants were verified as described by Haldimann and Wanner [2].

Plasmids were constructed as follows. To construct pNK11, *hipA* was PCR amplified with primers hipA-AccUP and hipA-CPae and inserted into pBAD33 using Acc65I and PaeI. To construct pNK12, *hipB* was PCR amplified with primers hipB-AccUP and hipB-EcoRDWN and inserted into pBRlacItac using Acc65I and EcoRI.

To construct pNK31, a part of the *relBEF* operon was PCR amplified with primers T7-AccEND and relE-R81A/R83A-Pae-HisDWN and inserted into pBAD33 using Acc65I and PaeI, followed by deletion of the PauI-MluI fragment of the plasmid.

To construct pNK32, a part of the *relBEF* operon was PCR amplified with primers T7-AccLL and relE-R81A/R83A-Pae-HisDWN and inserted into pBAD33 using Acc65I and PaeI, followed by deletion of the PauI-MluI fragment of the plasmid.

To construct pNK33, a part of the *relBEF* operon was PCR amplified with primers T7-Acc446 and relE-R81A/R83A-Pae-HisDWN and inserted into pBAD33 using Acc65I and PaeI, followed by deletion of the PauI-MluI fragment of the plasmid.

To construct pVK11, *relE* was PCR amplified with primers relE-XbaUP and relE-SalDWN and inserted into $pBAD22_{CM}$ using XbaI and SalI, followed by deletion of the XbaI-NheI fragment of the plasmid.

To construct pTM11, the fragment containing Ptac promoter and *gfp* gene of pET-GFPmut2 [3] was firstly inserted into pET19b using PaeI and XhoI. Then, the SspI-BspI fragment of the intermediate plasmid was inserted into the SmaI site of pAH162.

Luria-Bertani (LB) broth and LB agar plates were used for growth. Antibiotics were used at the following concentrations: ampicillin, 30 μg ml^{-1} or 100 μg ml^{-1}; chloramphenicol, 50 μg ml^{-1}; and kanamycin, 25 μg ml^{-1}. Liquid cultures were grown with aeration at 37°C.

RNA extraction using hot phenol

Cells were rapidly collected by centrifugation for 2 min at 8000 x g and frozen in liquid nitrogen. Frozen cells were stored at -80°C. For RNA isolation cells were resuspended in 600 µl of ice-cold 1x TEN solution (10 mM Tris pH 7.5, 1 mM EDTA, 100 mM NaCl) and added quickly to the 600 µl preheated phenol-1% SDS solution (pH 4.5). Samples were mixed gently by inverting tubes a few times, incubated for 5 min at 67°C, and were re-inverted after each minute during the incubation. Lysates were centrifuged for 10 min at 4°C at 15 700 × g. Since here, all preparations were done on ice. 450 µl of aqueous phase was collected and extracted with 600 µl of phenol:chloroform, mixed vigorously on vortex, centrifuged as above and further extracted with 500 µl of chloroform. After centrifugation for 1 min at 4°C at 15 700 × g, 350 µl of aqueous phase was allocated, 0.1 volumes of 3M RNase free sodium acetate (pH 5.2) was added, and RNA was precipitated with 1.2 volumes of ice-cold isopropanol during 30 min incubation at -20°C. Tubes were then centrifuged for 40 min at 15 700 × g, RNA pellets were washed with 700 µl of 75% ethanol and centrifuged for 5 min at 12 000 × g. Pellets were air dried at room temperature for 10 min, dissolved in ddH_20 and stored at -80°C.

Supplementary figures

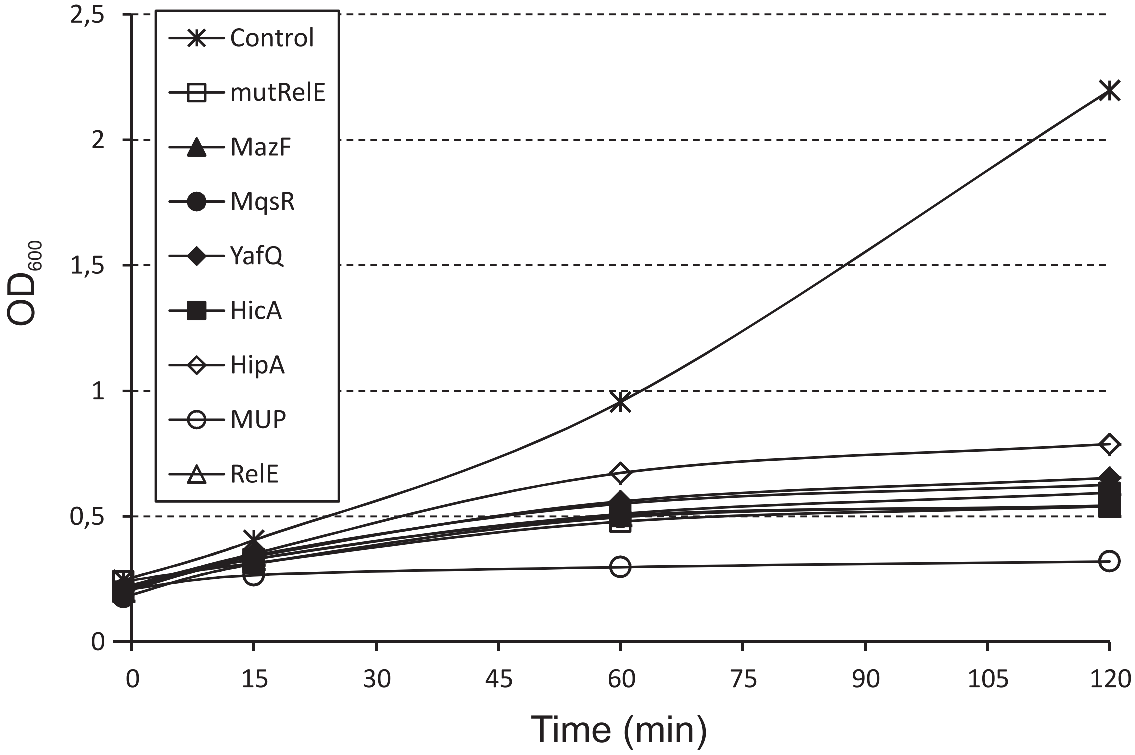

Fig. S1. Growth of the cultures used for extraction of RNA. RNA from these cultures was analyzed by northern hybridization (Fig. 1, 2 and S2) and primer extension (Fig. 4 and S4). mutRelE refers to the culture that contained plasmid pVK11 bearing the *relE* gene with synonymous mutations. RelE refers to the culture that contained pKP3035.

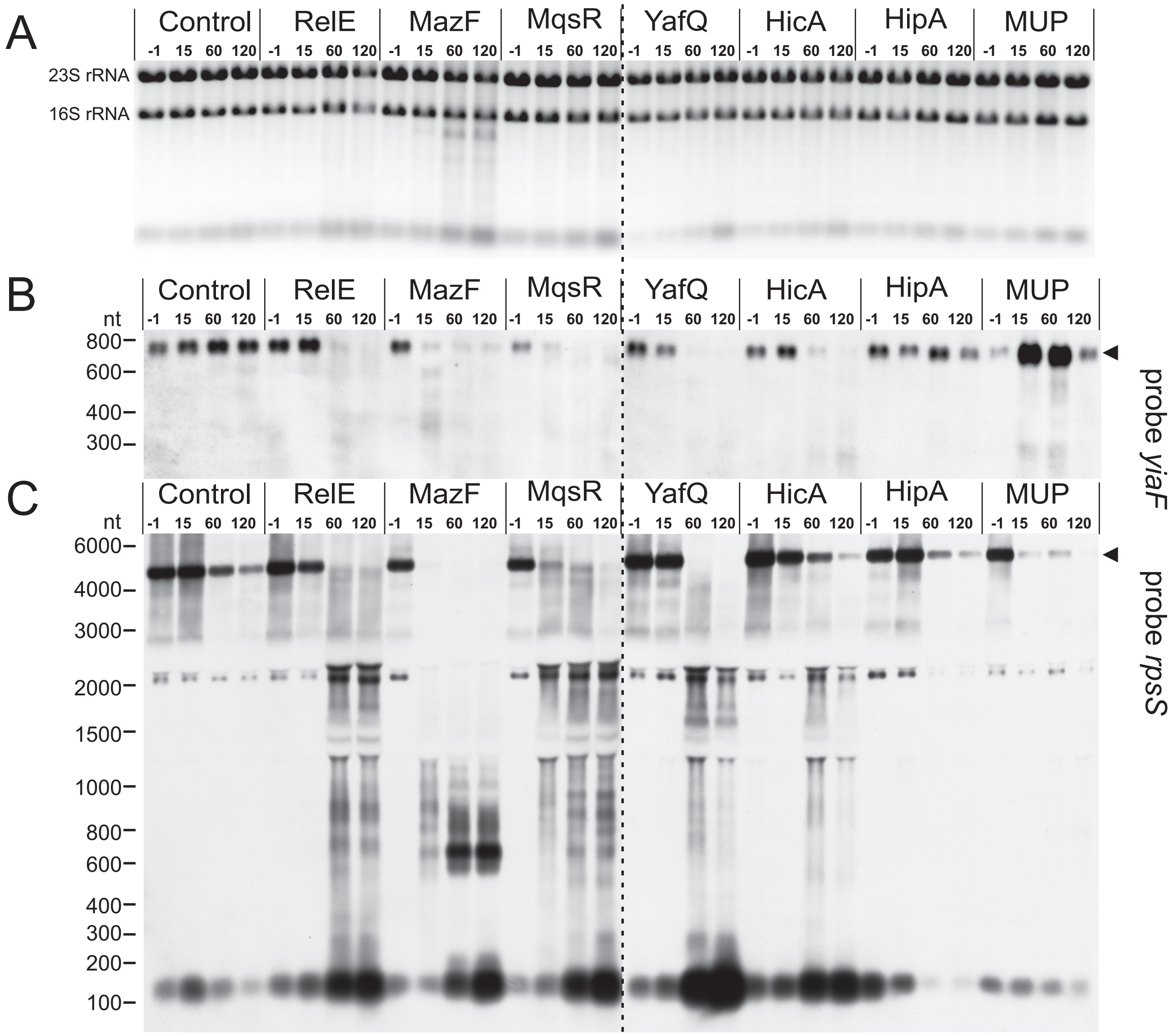

Fig. S2. Northern analysis of *yiaF* and *rpsS* transcription in response to expression of different toxins. The same filters as in Fig. 1 were hybridized with oligoprobes yiaF (B) and rpsS (C). The full-length *yiaF*, and S10 ribosomal protein operon transcripts are marked by arrowhead (◄). Ethidium bromide staining of 6 μg amounts of extracted RNA is shown to demonstrate equivalent RNA loading and intactness of samples (A).

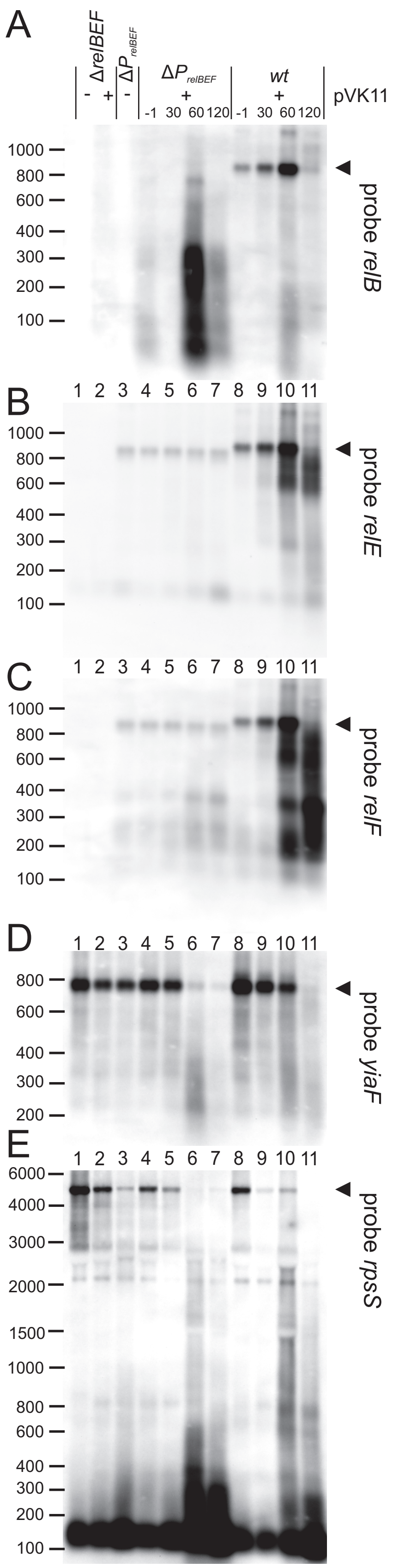

Figure S3. Northern analysis of transcription of the *relBEF* operon lacking its native promoter in response to ectopic expression of RelE.

Probes relB (A), relE (B), relF (C), yiaF (D), and rpsS (E) were used for hybridization. The full-length *relBEF*, *yiaF*, and 10S transcripts are marked by arrowheads (◄)

Lane 1: BW25113 Δ*relBEF* is a control for hybridization signal specificity; lane 2: BW25113 Δ*relBEF* containing plasmids pVK11 for RelE expression and pKP3033 for RelB expression. The lack of a hybridization signal in these lanes on panels A-C confirms that probes relB, relE and relF hybridize specifically to the *relBEF* mRNA and do not cross-hybridize to other RNA molecules of *E. coli*. The lack of hybridization signal in lane 2 panel B shows that probe relE does not cross-hybridize to the transcript of *relE* containing synonymous substitutions, which was expressed from pVK11 for 30 min.

Lane 3, BW25113 ΔP_{relBEF}; lanes 4-7: BW25113 ΔP_{relBEF} containing plasmids pVK11 for RelE expression and pKP3033 for RelB expression. Samples for RNA extraction were taken before RelE induction (lane 4) and 30, 60, and 120 min after RelE induction (lanes 5-7, respectively).

Lanes 8-11, BW25113 containing plasmid pVK11. Samples for RNA extraction were taken before RelE induction (lane 8) and 30, 60, and 120 min after RelE induction (lanes 9-11, respectively).

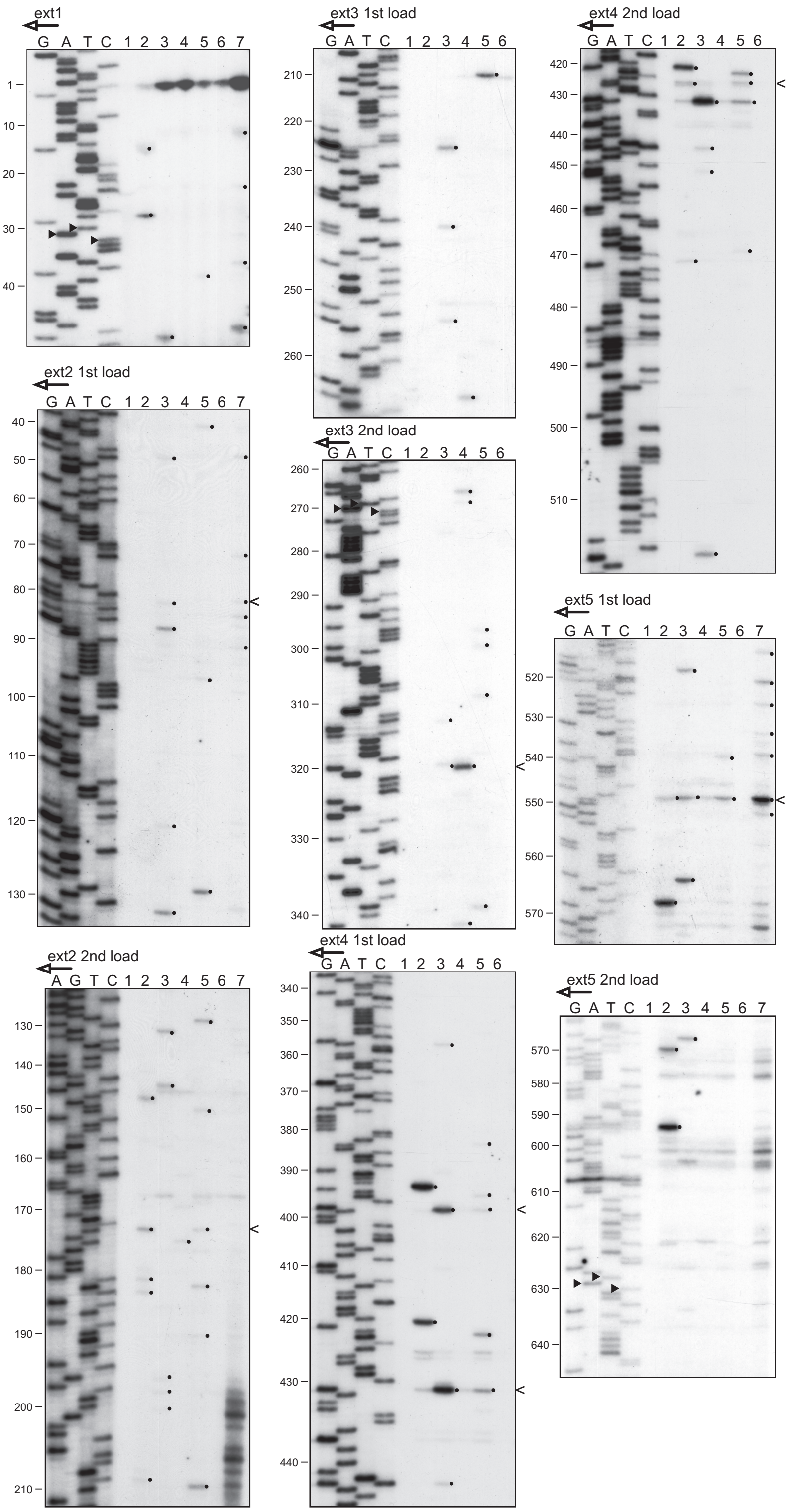

Fig. S4. Primer extension mapping of cleavage of the *relBEF* mRNA. The same RNA samples that were analyzed by northern blotting (Fig. 1) were subjected to primer extension analysis. Lane 1 (control) corresponds to an RNA sample taken at time 0 from an uninduced culture. Lanes 2-7 correspond to RNA samples taken 60 min after induction of MazF, MqsR, YafQ, HicA, HipA, and RelE, respectively. Primers ext1 to ext5 (Fig. 5, Table S2) were used in extension and sequence reactions (primer ext3 was not used for the RelE sample). The sequence is numbered starting at position +1 of the full-length *relBEF* mRNA. Filled triangles indicate start codons for *relB*, *relE*, and *relF*. Extension stops shown in Fig. 5 and Table S3 are marked with black dots. Arrowheads (<) on the left of the panels indicate extension stops that occurred in response to several over-produced toxins.

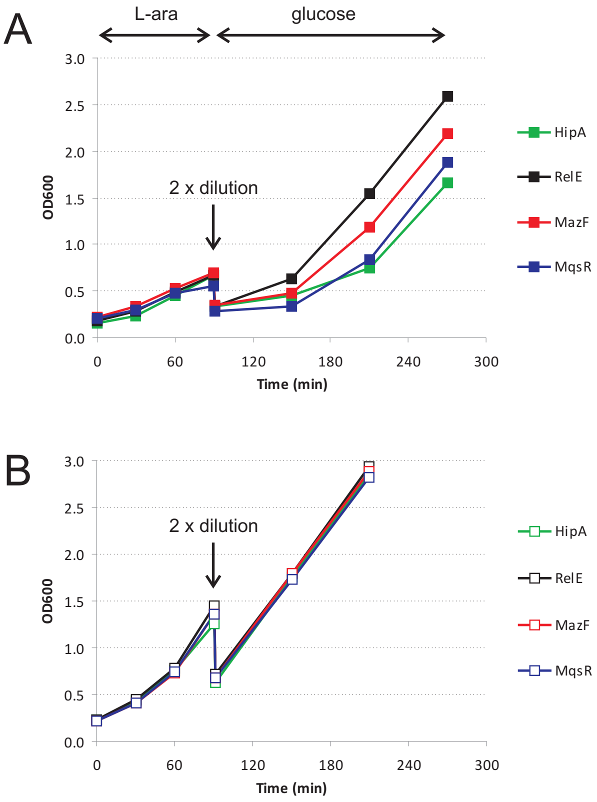

Figure S5. Growth of bacteria for monitoring recovery from transient expression of toxins. Cultures of BW25113-Ptac-GFP contained pKP3035 for RelE expression, pSC3326 for MazF expression, pNK11 for HipA expression, and pTX3 for MqsR expression. Cultures were grown in LB containing 0.2% glucose and expression of GFP was induced by adding 1mM IPTG for 2.5 h. At time 0, bacteria were transferred into LB supplemented with 1mM L-arabinose to induce toxins (A). Control cultures were grown in LB without arabinose (B). After 90 min, the cells were transferred into LB containing 0.2% glucose. Samples for FACS analysis were collected at 30 min intervals.

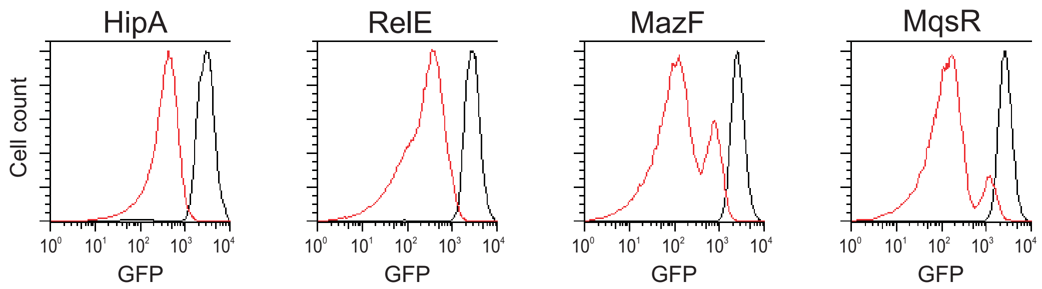

Figure S6. Growth resumption after transient production of toxins.
Growing cells of BW25113-Ptac-GFP, containing pKP3035 for RelE expression, pSC3326 for MazF expression, pNK11 for HipA expression, and pTX3 for MqsR expression, were filled with green fluorescent protein and transferred to LB supplemented with 1mM L-arabinose to induce toxins. After 90 min, the media were replaced with LB containing 0.2% glucose to repress toxin expression and enable regrowth. Histograms show the amount of GFP per cell at the moment of toxin induction (black) and after 150 min (red).

Supplementary tables

Table S1. Strains and plasmids used in this study

Strain or plasmid	Genotype	Reference
**Strains**		
BW25113	*lacI*q *rrnB3 _lacZ4787 hsdR514* Δ(*araBAD*)*567* Δ(*rhaBAD*)*568 rph-1*	[2]
BW25113 Δ*relBEF*	BW25113 Δ*relBEF*::*kan*	This study
BW25113 ΔP*relBEF*	BW25113 ΔP*relBEF*::*kan*	This study
BW25113 Δ*lon*	BW25113 Δ*lon*::*kan*	Keio collection [4]
BW25113 Δ*ppk*	BW25113 Δ*ppk*::*kan*	Keio collection [4]
BW25113 Δ*mazEF*	BW25113 Δ*mazEF*::*kan*	[5]
BW25113 Δ*mqsRA*	BW25113 Δ*mqsRA*::*kan*	[5]
BW25113 Δ*clpPX*Δ*lon*	BW25113 Δ*clpPX*Δ*lon*::*kan*	This study
BW25113 Δ*hslVU*	BW25113 Δ*hslVU*::*FRT*	[6]
BW25113 Δ*hslVU*Δ*clpPX*Δ*lon*	BW25113 Δ*clpPX*Δ*lon*::*kan*	This study
BL21(DE3)	B F^- *dcm ompT hsdS*(r_B^- m_B^-) *gal* λ(DE3 [*lacI lacUV*5-T7 gene 1 *ind*1 *sam*7 *nin*5])	Stratagene
BW23473	Δ*(argF-lac)*169 Δ*uidA3::pir*$^+$ *recA1 rpoS396*(Am) *endA9*(del-ins)*::FRT rph-1 hsdR*514 *rob*-1 *creC*510	[2]
BW25113-Ptac-GFP	BW25113 *att*φ80::pTM11	This study
**Plasmids**		
pKD13	*bla* FRT *aph* FRT PS1 PS2 oriR6K	[1]
pKD46	*bla* P_{BAD}-*gam bet exo* pSC101 oriTS	[1]
pCP20	*bla cat cI*857 λP_R-*flp* pSC101 oriTS	[1]
pBAD33	*cat araC* P_{BAD} pACYC184 ori	[7]
pBAD22$_{CM}$	*cat araC* P_{BAD} pBR322 ori	[8]
pET28a(+)	*kan* T7 expression vector	Novagen
pOU82	R1 replicon; *bla lacZYA*	[9]
pBRlacItac	*bla lacI*q P*tac* pBR322 ori	[10]
pKP3035	pBAD33-*relE*	[11]
pKP3033	pKP219-*relB*	[11]
pSC3326	pBAD33-*mazF*	[11]
pSC228	pNDM220-*mazE*	[11]
pMJ221	mini R1; *bla lacI*^{q} $P_{A1/O4/O3}$::*hicA*	[12]
pMJ331	pBAD33-*hicB*	[12]
pUHE *dinJ*	pUHE 25-2(*cat*)- *dinJ*	[13]
pBAD-*yafQ*	pBAD30- *yafQ*	[13]
pTX3	pBAD33-*mqsR*	[5]
pAT3	pBRlacItac-*mqsA*	[5]
pNK11	pBAD33-*hipA*	This study
pNK12	pBRlacItac-*hipB*	This study
pNK31	pBAD-PT7-*relBrelE*(R81A/R83A)	This study
pNK32	pBAD-PT7-ll-(+28)*relBrelE*(R81A/R83A)	This study
pNK33	pBAD-PT7-(+148)*relBrelE*(R81A/R83A)	This study
pVK11	pBAD22$_{CM}$ -*relE-synonymous*	This study
pAH162	oriR6Kγ *att*φ80 *tetA*	[2]
pAH123	*int* φ80 *LAMcI857*(ts) *bla* repA101(ts) oriR101	[2]
pET-GFPmut2	pET41A-Ptac-*GFPmut2*	[3]
pTM11	pAH162-Ptac-*GFPmut2*	This study

Table S2. Oligonucleotides used in this study

Oligonucleotide	Sequence (5'→3')
**Knock-out primers**	
relBEF promKO	TAAGAACGCGCTTTAAGTTCATCGTCAATACGCAGGTTAATGCTACCCATATTC CGGGGATCCGTCGACC
relBEF H1	AGCGGACAGTGATCACCGTTCTTACGACTACTTTCTGACTTCCTTCGTGAGTGT AGGCTGGAGCTGCTTC
relBEF H2	GGATGCCTGCCACATCAGAGGTGGCGAGGGATTTCTCCCCCGCCGGGTCTATTC CGGGGATCCGTCGACC
clpP P1	TGTCATGAATTTTGCATGGAACCGTGCGAAAAGCCTCTTTCGGTGTTAGCGTGT AGGCTGGAGCTGCTTC
lon P4	AAGCCCGAATTAGCCTGCCAGCCCTGTTTTTATTAGTGCATTTTGCGCGAATTCC GGGGATCCGTCGACC
**Knock-out control primers**	
relF DWN	CGCAGTTCTTCGATGTTC
relBEF UP	CGAGCTGACCTGCACAATAC
lon DWN	CGATCCGCCATCTAACTTAG
clpP UP	TTCGCACAAAGGCCCGTCAC
**Other PCR primers**[a]	
hipA-AccUP	CGGGGTACCAGGAGATATGGAATA**ATG**CCTAAACTTGTCACTTGGATG
hipA-CPae	ACATGCATGC**TCA**CTTACTACCGTATTCTC
hipB-AccUP	CGGGGTACCTAAGGAGATATATGGAATA**ATG**ATGAGCTTTCAGAAGATCTATA
hipB-EcoRDWN	CCGGAATTC**TTA**CCACTCCAGATTTTGCTGT
T7-AccEND[b]	CGGGGTACCTAATACGACTCACTATAGACATTTGTAATTACAAGAGG
T7-AccLL[b]	CGGGGTACCTAATACGACTCACTATAGATGGGTAGCATTAACCTGCGT
T7-Acc446[b]	CGGGGTACCTAATACGACTCACTATAGACAATGAACGCTTGCCGTTC
relE-R81A/R83A-Pae-HisDWN	ACATGCATGC**TCA**GTGGTGATGGTGATGATGGAGAATGCGTTTGACCGCCTCG CTATATACTTCCGATCGTTCTGCTTTCCCAAC
relE-XbaUP[c]	GGTATCTAGACGTGTGACGCTGGATGAACTCTGATGGCGTATTTCTGGATTTT GACGAGCGcGCttTgAAaGAgTGGCGtAAatTaGGgagcACcGTtCGcGAgCAGTTGAAA AAGAAGCTGGT
relE-SalDWN	CGAGGTCGACTCAGAGAATGCGTTTGAC
**Hybridization probes**	
relB	GAACGCGCTTTAAGTTCATCGTCAATACGCAGGTTAATGCTACCC
relE	TGTTCACGTACCGTCGAGCCCAGCTTTCGCCATTCCTTTAGTGCC
relF	ACCTCGCAGAGGTCTTTCCTCGTTACCAGTGCCGTCACTATGACG
mazE	GGTAAGCCGATACGTACCCGATATGGGCGATCTGATTTGGGTTGA
mazF	CTGTTCCTTTCTTCGTTGCTCCTCTTGCCCGCCAGGCGATACTTT
mqsA	CGAAGCCCGAAATGCCTTTACTTGCGCCATGAAAGCATCTGACTC
mqsR	GATAAACCTGGCCTGTAACAAGCCTGGGTCTGTAAACATCCTGCC
dinJ	TCAGCCCCATCCCGGCCAGTACGTCCGCTGCCTGATTCTTCAGAT
yafQ	GACGCTTTTGTGCAAGTTTTACATCCTTTGAATATTGTCCCGAGT
hicA	ATGGTTGCTGCCATTCGCTACATCGACGCCCTGAGATTCGAGCCA
hicB	AGTGATCGTGACTATTTAATGGCGAAGGTAAAGGGATAAGCTCGT
yefM	CTGACGCGCTTCGCTGTAGCTAATTGTACGCATGTCAATCTCCTC
yoeB	CAGACGGTGCTCCTCTGTAATGCGTCGGGACCAGAAACCTGACAA
prlF	CCTTTGATTCAGTGGTCAGTACAGCGTGAGAGCGAGCATTAGCGG
yhaV	CCTAACGACTTGCCATGACGGAATGCCGGTGATGATGGATTGACC
yiaF	TTCTGATCGGCAGTCAGGGTTGGTAGACGTTCACCGCTACGCATC
rpsS	GGGCTTCTTGTCTCCGCTTTCCACCGCTTTCTCTACCTTCTTCAG
**Extension primers**	
ext1	TACTCGAGCATGAGACGAAG
ext2	CGCCATCAGAGTTCATCCAG
ext3	CATACCACGGAGCTTGTTTG
ext4	CGGTGCGAAACAGAGATGTC
ext5	CCGGTTCGGATTCGTACCTC

[a] Restriction sites in noncomplementary overhangs are underlined. Start and stop codons are in bold

[b] Underlined G marks the 5' end of the T7 transcript

[c] Mutated nucleotides in lowercase

Table S3. Cleavage sites of *relBEF* mRNA in vivo.

Toxin	Lane Figure S6	Position relative to the mRNA 5' end	Sequence 5'→3'
MazF	2	15	AAUUAˇCAAGA
(ˇAˇCA)		28	GUAAGˇACAUG
		148	CGCUGˇACAAU
		**174**	**AACAGˇACACU**
		182	CUCCUˇGAGUG
		184	CCUGAˇGUGAU
		209	GUGGAˇGAUAG
		394	GCAAAˇCAAGC
		421	UGUUAˇCAAGA
		472	AUAGAˇCGAGA
		**549**	**ACGCAˇUUCUC**
		569	CAUGAˇCAUCU
		594	GGUGAˇCACUU
MqsR	3	48	ACCUGˇCGUAU
(GˇCU)		**83**	**UACGCˇCGCGC**
		87	CCGCGˇCUUGA
		120	AAGCGˇCUUCG
		132	UCAUGˇCUCGA
		145	UAUCGˇCUGAC
		196	AGAUGˇCUGAA
		198	AUGCUˇGAACU
		200	GCUGAˇACUUG
		225	AACGGˇCUUCG
		240	CUAAGˇCCAGU
		255	UGACGˇCUGGA
		313	GAAUGˇGCGAA
		320	GAAAGˇCUGGG
		357	GAAGCˇUGGUU
		**399**	**CAAGCˇUCCGU**
		**431**	**UUAAGˇCUCCG**
		445	UCAGGˇCUAUC
		451	UAUCGˇCCUUG
		517	GAACGˇCUCGG
		**549**	**ACGCAˇUUCUC**
		564	CAAAGˇCAUGA
YafQ	4	176	CAGACˇACUCC
(AAˇA-G/A)		266	GAACUˇCUGAU
		269	CUCUGˇAUGGC
		320	GAAAGˇCUGGG
		341	GUGAAˇCAGUU
HicA	5	39	GUAGCˇAUUAA
		97	AAAAAˇUGGGU
		130	UCUCAˇUGCUC
		151	UGACAˇAUGAA
		**174**	**AACAGˇACACU**
		183	UCCUGˇAGUGA
		190	UGAUGˇAAGAU
		210	UGGAGˇAUAGU
		297	CGAGCˇGGGCA
		300	GCGGGˇCACUA
		309	AAAGGˇAAUGG
		339	ACGUGˇAACAG
		384	CCGGAˇUUGAA
		396	AAACAˇAGCUC

		**399**	**CAAGCˇUCCGU**
		423	UUACAˇAGAUU
		**431**	**UUAAGˇCUCCG**
		470	UUAUAˇGACGA
		540	GGCGGˇUCAAA
RelE	7	11	UUGUAˇAUUAC
		24	AGGUGˇUAAGA
		37	GGGUAˇGCAUU
		47	AACCUˇGCGUA
		71	AAAGCˇGCGUU
		**83**	**UACGCˇCGCGC**
		87	CCGCGˇCUUGA
		91	GCUUGˇAAAAA
		513	AAGAGˇAACGC
		521	GCUCGˇGAAGU
		527	AAGUAˇUAUAG
		534	UAGCGˇAGGCG
		540	GGCGGˇUCAAA
		**549**	**ACGCAˇUUCUC**
		552	CAUUCˇUCUGA

Specific cutting sequences of MazF (ˇAˇCA) and MqsR (GˇCU) are underlined. Sites where RNA is cleaved in response to several over-produced toxins are in bold.

References

1. Datsenko KA, Wanner BL: **One-step inactivation of chromosomal genes in Escherichia coli K-12 using PCR products**. *Proceedings of the National Academy of Sciences of the United States of America* 2000, **97**(12):6640-6645.
2. Haldimann A, Wanner BL: **Conditional-replication, integration, excision, and retrieval plasmid-host systems for gene structure-function studies of bacteria**. *J Bacteriol* 2001, **183**(21):6384-6393.
3. Vimberg V, Tats A, Remm M, Tenson T: **Translation initiation region sequence preferences in Escherichia coli**. *BMC molecular biology* 2007, **8**:100.
4. Baba T, Ara T, Hasegawa M, Takai Y, Okumura Y, Baba M, Datsenko KA, Tomita M, Wanner BL, Mori H: **Construction of Escherichia coli K-12 in-frame, single-gene knockout mutants: the Keio collection**. *Molecular systems biology* 2006, **2**:2006 0008.
5. Kasari V, Kurg K, Margus T, Tenson T, Kaldalu N: **The Escherichia coli mqsR and ygiT genes encode a new toxin-antitoxin pair**. *J Bacteriol* 2010, **192**(11):2908-2919.
6. Hansen S, Vulic M, Min J, Yen TJ, Schumacher MA, Brennan RG, Lewis K: **Regulation of the Escherichia coli HipBA toxin-antitoxin system by proteolysis**. *PloS one* 2012, **7**(6):e39185.
7. Guzman LM, Belin D, Carson MJ, Beckwith J: **Tight regulation, modulation, and high-level expression by vectors containing the arabinose PBAD promoter**. *J Bacteriol* 1995, **177**(14):4121-4130.
8. Vazquez-Laslop N, Lee H, Neyfakh AA: **Increased persistence in Escherichia coli caused by controlled expression of toxins or other unrelated proteins**. *J Bacteriol* 2006, **188**(10):3494-3497.
9. Gerdes K, Larsen JE, Molin S: **Stable inheritance of plasmid R1 requires two different loci**. *Journal of bacteriology* 1985, **161**(1):292-298.

10. Ojangu EL, Tover A, Teras R, Kivisaar M: **Effects of combination of different -10 hexamers and downstream sequences on stationary-phase-specific sigma factor sigma(S)-dependent transcription in Pseudomonas putida**. *J Bacteriol* 2000, **182**(23):6707-6713.
11. Pedersen K, Christensen SK, Gerdes K: **Rapid induction and reversal of a bacteriostatic condition by controlled expression of toxins and antitoxins**. *Mol Microbiol* 2002, **45**(2):501-510.
12. Jorgensen MG, Pandey DP, Jaskolska M, Gerdes K: **HicA of Escherichia coli defines a novel family of translation-independent mRNA interferases in bacteria and archaea**. *J Bacteriol* 2009, **191**(4):1191-1199.
13. Motiejunaite R, Armalyte J, Markuckas A, Suziedeliene E: **Escherichia coli dinJ-yafQ genes act as a toxin-antitoxin module**. *FEMS Microbiol Lett* 2007, **268**(1):112-119.
